# Carbapenemase-Producing Non-Glucose-Fermenting Gram-Negative Bacilli in Africa, *Pseudomonas aeruginosa* and *Acinetobacter baumannii*: A Systematic Review and Meta-Analysis

**DOI:** 10.1155/2020/9461901

**Published:** 2020-11-03

**Authors:** Mizan Kindu, Lemma Derseh, Baye Gelaw, Feleke Moges

**Affiliations:** ^1^Department of Medical Microbiology, College of Medicine and Health Science, University of Gondar, Gondar, P.O. Box 196, Ethiopia; ^**2**^ Department of Epidemiology and Biostatistics Institute of Public Health College of Medicine and Health Sciences, University of Gondar, Gondar, P.O. Box 196, Ethiopia

## Abstract

**Background:**

Studies have reported that the existence of CP bacteria in Africa, but, in general, comprehensive data about the molecular epidemiology of CP organisms are limited. Therefore, this systematic review and meta-analysis expound the pooled prevalence of CP *P. aeruginosa* and CP *A. baumannii* clinical isolates in Africa. It also identified the diversity of carbapenemases or their encoding genes among the isolates in Africa. Lastly, the review observed the trends of these CP isolates in Africa.

**Methods:**

A comprehensive search was performed between July 2019 and October 2019 in the following databases: PubMed, Google Scholar, and African Journal online. The included articles were published only in English. The screening was done by two authors independently. The data extracted on Excel spreadsheet were transferred to STATA 11 software for analysis.

**Results:**

From a total of 1,454 articles searched, 42 articles were eligible. Most of the studies were conducted in the North Africa region. But there was no report from Central Africa. The pooled prevalence of CP *P. aeruginosa* and CP *A. baumannii* among the clinical specimens in Africa was 21.36% and 56.97%, respectively. OXA-23 and VIM were the most prevailing carbapenemase among *P. aeruginosa* and *A. baumannii,* respectively. The cumulative meta-analysis revealed a relative increment of the prevalence of CP *P. aeruginosa* over time in Africa but it showed a higher prevalence of CP *A. baumannii* isolates across years.

**Conclusion:**

The review revealed a high pooled prevalence of CP *A. baumannii* clinical isolates in Africa which needs urgent action. Moreover, the emergence of concomitant carbapenemases, especially OXA-23 +  NDM among CP *A. baumannii*, was also an alarming problem.

## 1. Introduction

Carbapenem-resistant (CR) *Acinetobacter baumannii* (CRAB) and CR *Pseudomonas aeruginosa* (CRPA) are among the top tier of the World Health Organization (WHO) list of antibiotic-resistant “priority pathogens” that pose the greatest threat to human health [1]. Infections with these resistant bacteria are a matter of national and international concern as they are an emerging cause of Hospital Acquired Infections (HAIs) that pose a significant threat to public health and responsible for hospital outbreaks worldwide [2]. Moreover, they are associated with high rates of morbidity and mortality, especially in patients with serious underlying disorders or patients admitted to the intensive care unit (ICU) [3].

Although carbapenem antibiotics were introduced to treat infection caused by bacteria resistant to penicillin, cephalosporins, and fluoroquinolones, the reliability of these antibiotics has been reduced because of resistance. Multiple mechanisms of carbapenem resistance have been identified including overexpression of efflux pumps, porin mutations, and enzymatic inactivation [4].

The production of an enzyme carbapenemase, having the ability to hydrolyze almost all beta-lactam antibiotics, has been the most critical mechanism that causes resistance to carbapenems [5]. Carbapenemases belong to classes A, B, and D of ambler classification beta-lactamases [6]. Carbapenemase that belongs to class A beta-lactamase utilizes serine for *β*-lactam hydrolysis that contains a wide variety of enzymes including KPC (*Klebsiella pneumoniae* carbapenemase = KPC-2 to KPC-13), IMI (Imipenem-hydrolyzing *β*-lactamase; IMI-1 to IMI- 3), and GES (Guiana extended spectrum; GES-1 to GES-20), with hydrolyzing activity against carbapenems. These enzymes are inhibited by clavulanic acid. The KPCs are the most frequently identified class A carbapenemases [5].

Likewise, these belong to class D beta-lactamases (oxacillinase = OXA-types) and also utilize the serine-active site for *β*-lactam hydrolysis. OXA enzymes (the name came since they hydrolyze isoxazolyl penicillins much faster than penicillins) were recently divided into 12 subgroups: OXA-23-like, OXA24/40-like, OXA-48, OXA-51-like, OXA-58-like, OXA-143-like, OXA-253, OXA-211, OXA-213, OXA- 214, OXA-229, and OXA-235 [7]. The OXA-23, OXA-24/40, OXA-48, and OXA-58 carbapenemases, which are mainly plasmid-encoded, and the OXA-51 carbapenemase, which is chromosomally encoded and intrinsic (naturally found) in *A. baumannii*. Class D enzymes are not inhibited by clavulanic acid or ethylenediaminetetraacetic acid (EDTA) [8].

On the contrary, carbapenemases found under class B beta-lactamases (also referred metallo-beta-lactamase = MBL) use active-site zinc to hydrolase all *β*-lactams except for aztreonam, a monobactam, and are susceptible to inhibition by EDTA. These enzymes include but are not limited to VIM (Verona integron-encoded metallo-beta-lactamase), GIM (German imipenemase), IMP, AIM-1 (Adelaide imipenemase 1), SIM (Seoul imipenemase), and NDM (New Delhi metallo-beta-lactamase) [9].

Studies have reported that the existence of carbapenemase-producing (CP) bacteria in Africa [10–13] but in general comprehensive data about the molecular epidemiology of CP organisms in Africa is limited. Therefore, this systematic review and meta-analysis expound the pooled prevalence of CPPA and CPAB clinical isolates in Africa. Moreover, it also systematically reviews the diversity of carbapenemases and their encoding genes among these CP clinical isolates in Africa. Lastly, the review observed the trends of these isolates in Africa.

## 2. Methods

### 2.1. Protocol Registration and Review Reporting

This systematic review and meta-analysis was registered at the international prospective register of systematic review and meta-analysis (PROSPERO) with the registration number of CRD42019147430. The results of this review were reported based on the Preferred Reporting Items for Systematic Review and Meta-Analysis (PRISMA) statement guideline [14].

### 2.2. Searching Strategy and Information Sources

To find potentially relevant articles, comprehensive searches were performed between July 2019 and October 2019 in the following databases: PubMed/MEDLINE, Google Scholar, and African Journal online. All searches were limited to articles written in English. The search was also supplemented by searching gray literature of observational study and reference lists of the eligible studies.

The search strings or terms were stemmed from the isolates of each individual. For *P. aeruginosa,* we used *P. aeruginosa*, carbapenemase-producing *P. aeruginosa,* class A carbapenemases, class B carbapenemases, Metallo-*β*-lactamase, class D carbapenemases, oxacillinase, and Africa. For *A. baumannii*, we used *A. baumannii*, carbapenemase-producing *A. baumannii*, class A carbapenemases, class B carbapenemases, Metallo-*β*-lactamase, class D carbapenemases, oxacillinase, and Africa. In the advanced searching databases, the searching strategy was built based on the abovementioned terms using the “Medical Subject Headings (MeSH)” and “All fields” by linking “AND” and “OR” Boolean operator terms as appropriate.

### 2.3. Inclusion and Exclusion Criteria

We included studies reporting the prevalence of CPPA and CPAB infection among clinical specimens recovered from human patients, cross-sectional studies, studies published in English, and studies that detected carbapenemase producers using phenotypic and confirmed through molecular methods.

Studies were excluded if they reported carriage rather than infection, conducted environments, and detected carbapenemase production only using the phenotypic method. Review articles, meta-analyses, case reports, case series, letters to the editors, correspondence, outbreak settings, and articles which were not fully accessible after at least two-email contact with the corresponding author were also excluded. The exclusion of these articles was because of the inability to assess the quality of articles in the absence of full text.

### 2.4. Quality Assessment

Three independent reviewers (MK, BG, and FM) examined the quality of the included studies. Since all the included studies were cross-sectional in nature, the quality of each article was assessed using the Joanna Briggs Institute (JBI) critical appraisal tool prepared for cross-sectional studies [15]. The quality scales of primary studies were considered as low risk for both systematic review and meta-analysis if the studies got 50% and above.

### 2.5. Data Extraction Process

Two reviewers (MK and BG) independently extracted data including the name of the first author, year of publication and sample collection, the study design, the total number of each bacterial isolates identified, the number of CP isolates, the number and type of the carbapenemase enzyme or encoding genes detected for each specific bacterial isolate, the number of CR isolates from antibiotic resistance susceptibility test result, and countries in which the study was done. When disagreement occurred in the process of data extraction, it was resolved by discussion and consensus. The third and the fourth authors (FM and LG) were consulted when consensus could not be achieved.

### 2.6. Outcome Measures

The primary outcome measure was the prevalence of CP *P. aeruginosa* and *A. baumannii* isolates. The prevalence of carbapenemase producers for each of the isolates was calculated independently as follows. The first was by dividing the total number of CP isolates by the total number of the identified isolates. The second was calculated by dividing the total number of CP isolates by their total number of bacterial isolates resistance to carbapenem through antibiotic susceptibility testing. The secondary outcome was the identification of the detected carbapenemase enzymes or their encoding genes for each specific bacterial isolates. The last outcome was also the time trend analysis for the prevalence of CP isolates in Africa.

### 2.7. Data Processing and Analysis

The data extracted in Microsoft Excel format was analyzed using STATA Version 11 statistical software. The existence of heterogeneity among studies was examined by the forest plot as well as the *I*^2^ heterogeneity test, in which 0–40%, 50–60%, 50–90%, and 75–100% represented low, moderate, substantial, and considerable heterogeneity, respectively [16, 17]. The *I*^2^ heterogeneity test ≥50% and a *p* value of <0.05 assured the presence of heterogeneity. Thus, the DerSimonian–Laird random-effects model was employed [18]. To identify the influential studies that resulted in variation, a sensitivity analysis was carried out using the “metaninf” command [19]. Similarly, subgroup analyses were also employed by assuming the region as grouping variables and sources of variation.

Furthermore, using the “metafunnel” command [20] and objectively by Egger's regression test, publication bias was detected [20]. Accordingly, asymmetry of the funnel plot and/or statistical significance of Egger's regression test (*p* value<0.05) was suggestive of publication bias [21]. Finally, all statistical interpretations were reported based on the 95% CI.

## 3. Results

### 3.1. Characteristics of the Included Studies

**Figure 1:**
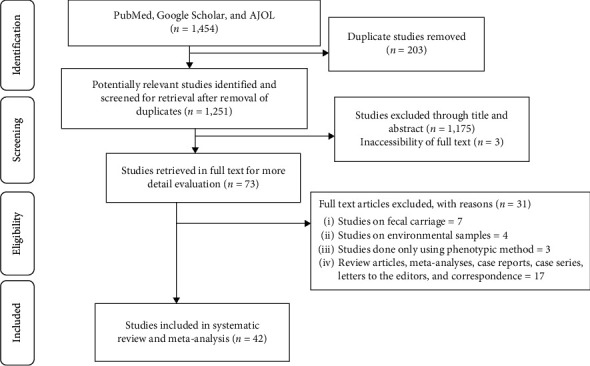
Flow chart shows the study selection process.

During our literature search, 1,454 potentially relevant articles were identified, of which 203 duplicated articles were excluded (Figure 1). All titles and abstracts of the remaining 1,251 articles were screened, resulting in the exclusion of 1,175 publications. Moreover, 3 articles were also excluded due to the inaccessibility of full text. The remaining 73 articles underwent a second screening based on the inclusion and exclusion criteria of the review and 31 of them were excluded. Finally, 42 articles were included in the systematic review and meta-analysis (Figure 1).

Among the articles, those which fulfill eligibility criteria of this systematic review and meta-analysis, most of the studies reporting molecular identification of CPPA and CPAB were conducted in the North Africa region (*n* = 32; 76.19%). In this region, mainly Egypt (*n* = 17) conducted the detection CP bacterial isolates using molecular methods followed by Algeria (*n* = 8), Morocco (*n* = 2, Tunisia (*n* = 2), Sudan (*n* = 2), and Libya (*n* = 1). The remaining 10 studies (23.81%) were carried out in other parts of African regions such as Ethiopia (*n* = 1), Uganda (*n* = 2), Tanzania (*n* = 1), Ghana (*n* = 1), Nigeria (*n* = 1), and South Africa (*n* = 4) (Table 1). However, there was no study from Central Africa.

In this systematic review and meta-analysis, clinical samples were collected from 2007 to 2018 G.C. from the abovementioned African countries. Moreover, some of the studies were conducted by focusing only on a single isolate either on *“A*. *baumannii”* (*n* = 13) or on “*P*. *aeruginosa”* (*n* = 18). The remaining (*n* = 11) involved multiple Gram-negative bacterial isolates (Table 1).

### 3.2. Carbapenemase-Producing *P*. *aeruginosa* in Africa

#### 3.2.1. Study Characteristics

According to the eligibility criteria of this systematic review and meta-analysis, a total of thirty studies reporting CPPA were included. Most of the studies (*n* = 24) reporting CPPA isolates were conducted in North Africa, majorly from Egypt followed by Algeria. The remaining six were carried out in other parts of the African region such as Uganda, Tanzania, Ghana, Nigeria, and South Africa. There was no report from Central Africa (Table 1).

#### 3.2.2. Geographical Distribution of Carbapenemase-Producing *P. aeruginosa* in Africa

**Figure 2:**
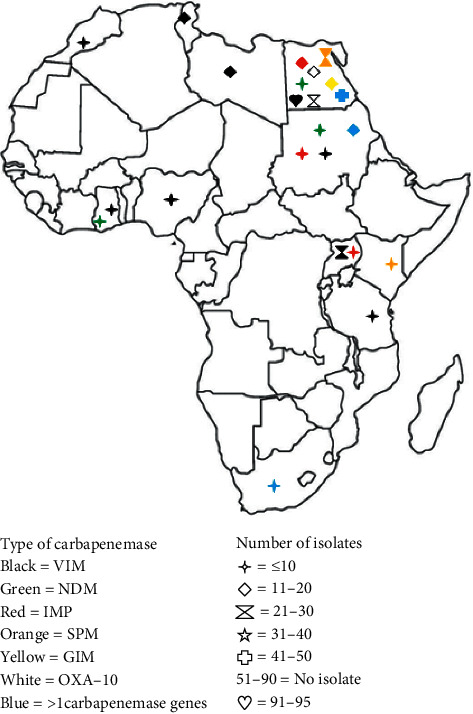
Geographical distribution CPPA isolates in Africa from 2007 to 2018 G.C.

The prevalence of CPPA among total isolates identified among all studies in North Africa ranged from 0% (0/9 [22], 0/80 [24]) to 84% (28/33) [37] while it ranged from 2.3% (25/1077) [52] to 21.4% (9/42) [51] in East Africa. Among carbapenemase enzymes, VIM was highly detected along with CPPA isolates in the reported African countries. OXA-10, IMP, SPM, NDM, GES, and GIM carbapenemases were also detected among the isolates (Figure 2). As indicated in Figure 2, all of the identified carbapenemase types among *P. aeruginosa* isolates were detected in Egypt. VIM-producing *P. aeruginosa* isolates were also observed in the majority of African countries (Figure 2). A total of 66 CPPA isolates were carbapenemase coproducers, carrying genes encoding more than one carbapenemase (Table 1).

#### 3.2.3. Pooled Prevalence of Carbapenemase-Producing *P. aeruginosa* in Africa

**Figure 3:**
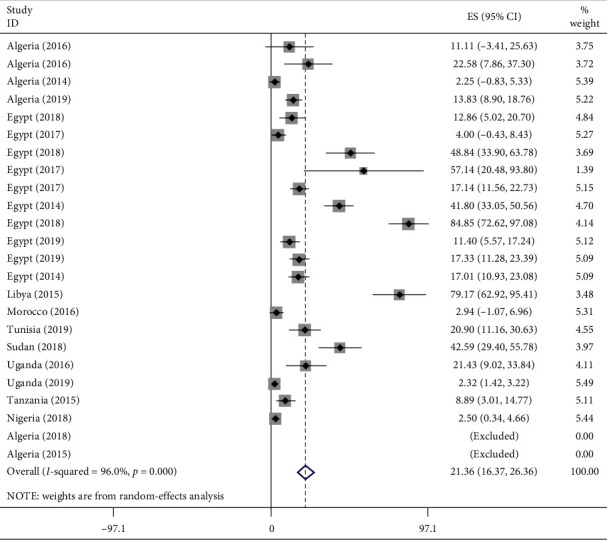
Forest plot for the pooled prevalence of CPPA isolates in Africa from 2007 to 2018 G.C.

From a total of 2,973 *P. aeruginosa* isolates collected from patients between 2007 and 2018 in Africa, the pooled prevalence of CPPA was 21.36%. As shown in Figure 3, there is a statistically significant considerable heterogeneity with I^2^ = 96% and *p*value of less than 0.001. The pooled result indicates further applying of subgroup and sensitivity analysis for detecting the most influential studies and identifying the source of heterogeneity, respectively. Moreover, among CRPA isolates, the pooled prevalence of CPPA was 53.21% (*I* = 97.0%, *p* < 0.001) (Figure S1) in Supplementary Materials.

#### 3.2.4. Subgroup Analysis

**Figure 4:**
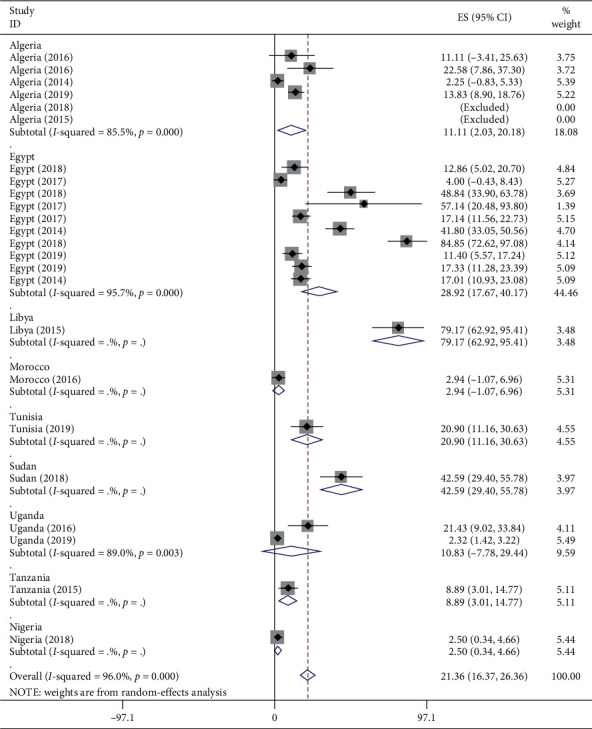
Subgroup analysis for the pooled prevalence of CPPA isolates in Africa by country from 2007 to 2018 G.C.

The results of the subgroup analysis done by considering both regions (Figure S2) in Supplementary Materials and country variables showed that there was still considerable heterogeneity (Figure 4).

#### 3.2.5. Sensitivity Analysis

**Figure 5:**
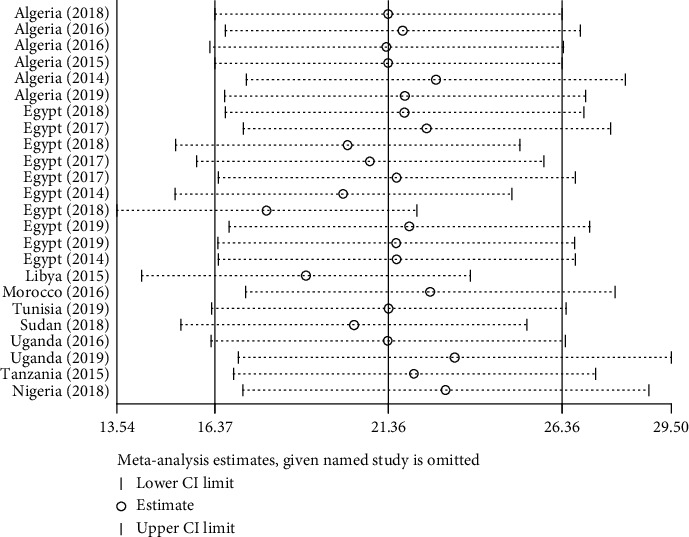
Sensitivity analysis for the pooled prevalence of CPPA in Africa from 2007 to 2018 G.C.

The result of the sensitivity analysis showed that there was no outlier study, which had an impact on the overall estimation (Figure 5).

#### 3.2.6. Publication Bias

**Figure 6:**
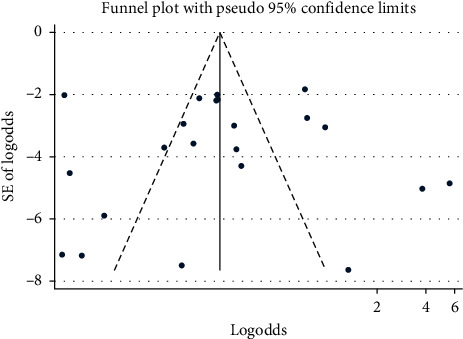
Funnel plot for publication bias of pooled prevalence of CPPA in Africa from 2007 to 2018 G.C.

A funnel plot showed a symmetrical distribution (Figure 6). Egger's regression test *p* value was also 0.909, which indicated no evidence for publication bias (Table S3) in Supplementary Materials.

#### 3.2.7. Time Trend Analysis of Carbapenemase-Producing *P. aeruginosa* in Africa

**Figure 7:**
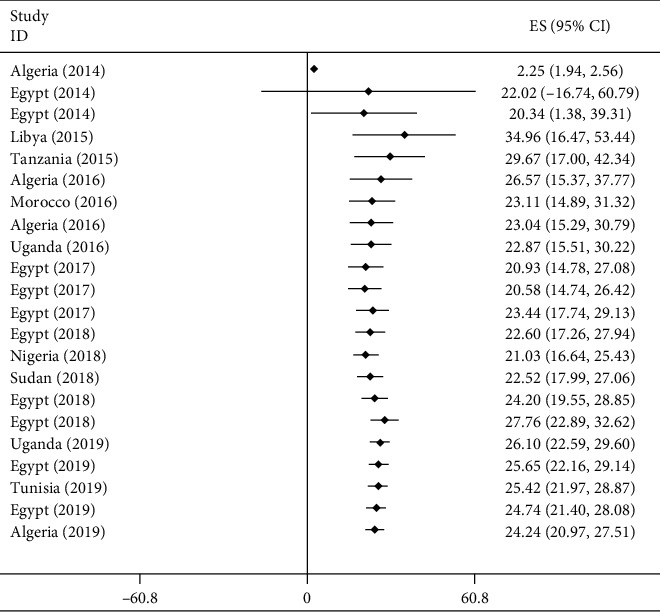
Cumulative meta-analysis of CPPA between 2014 and 2019 G.C. in Africa.

The cumulative meta-analysis result showed a relative increment trend of CPPA isolates over time in Africa among the studies published from 2014 to 2018 (Figure 7).

### 3.3. Carbapenemase-Producing *A. baumannii* in Africa

#### 3.3.1. Study Characteristics

A total of 23 studies that fulfilled the inclusion criteria of this systematic review and meta-analysis reporting CPAB were involved. Except for seven studies that were conducted in Uganda (2/23), Ethiopia (1/23), Ghana (1/23), and South Africa (3/23), the rest of the studies (*n* = 16) were done in North Africa. The majority of studies reporting CPAB in North Africa were from Egypt (8/16) followed by Algeria (4/16) (Table 1).

#### 3.3.2. Geographical Distribution of Carbapenemase-Producing *A. baumannii* in Africa

**Figure 8:**
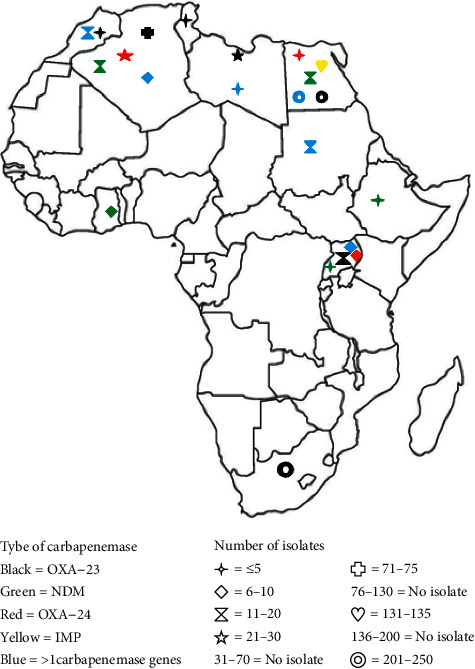
Geographical distribution of CPAB isolates in Africa from 2007 to 2017 G.C.

The lowest prevalence of CPAB among the total isolates identified in Africa was 4.7% (21/448), reported from Uganda [52], whereas the higher prevalence was 100% (7/7), reported from a study conducted in Algeria [23]. As shown in Figure 8, the most prevailing carbapenemase enzymes produced by *A. baumannii* isolates were OXA-23. Likewise, other carbapenemases such as IMP, NDM, and OXA-24 were also detected. But OXA-48, OXA-58, and GES carbapenemases were reported rarely (Table 1). Three or more types of carbapenemases were reported from Algeria, Egypt, Libya, and Uganda (Figure 8). Likewise, a high number of OXA-23-producing *A. baumannii* isolates were reported from Algeria, Egypt, and South Africa. Moreover, OXA-23 carbapenemases had also wider distribution along the studied African countries (Figure 8). The number of *A. baumannii* clinical isolates that coproduced more than one carbapenemase type was 273. Moreover, CPAB isolates coproducing OXA-23 + NDM had wider distributions (Table 1).

#### 3.3.3. Pooled Prevalence of Carbapenemase-Producing *A. baumannii* in Africa

**Figure 9:**
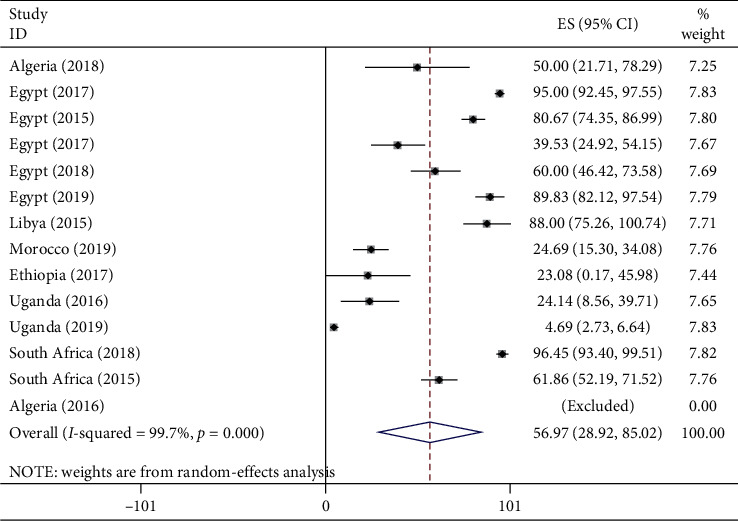
Forest plot for the pooled prevalence of CPAB isolates in Africa from 2007 to 2017 G.C.

Among the total of 1,435 *A. baumannii* isolates collected from 2007 to 2017 in Africa, the pooled prevalence of carbapenemase producers was 56.97% with I^2^ 99.7% and *p* value of less than 0.001 (Figure 9). The pooled prevalence of CPAB isolate among CRAB isolates was also 86.11% (*I*^2^ = 92.4%, *p* < 0.001) (Figure S4) in Supplementary Materials.

#### 3.3.4. Subgroup Analysis

**Figure 10:**
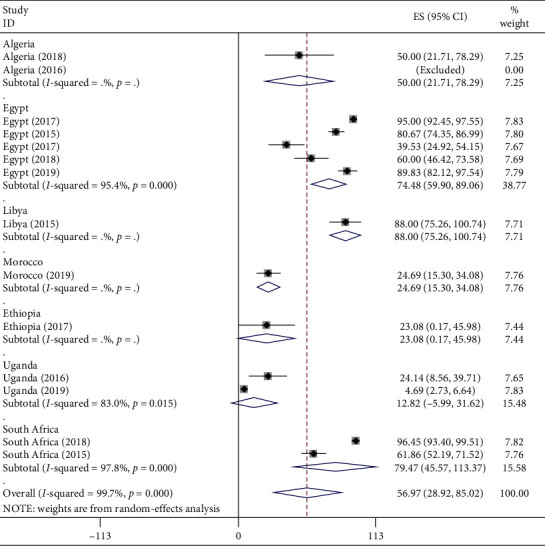
Subgroup analysis for the pooled prevalence of CPAB in Africa by country from 2007 to 2017 G.C.

The results of the subgroup analysis done by considering both regions (Figure S5) in Supplementary Materials and country variables (Figure 10) showed still that heterogeneity was considerable.

#### 3.3.5. Sensitivity Analysis

**Figure 11:**
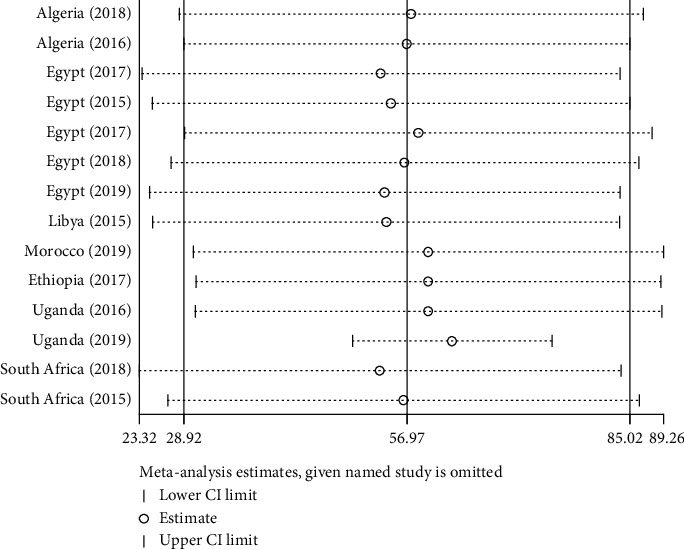
Sensitivity analysis for the pooled prevalence of CPAB in Africa from 2007 to 2017 G.C.

As shown in Figure 11, the prevalence of CPAB isolates from each study was within the confidence interval limit. This shows that they do not have any influence on the overall estimation.

#### 3.3.6. Publication Bias

**Figure 12:**
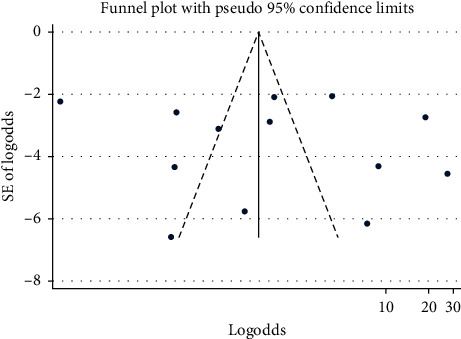
Funnel plot for publication bias of pooled prevalence of CPAB in Africa from 2007 to 2017 G.C.

In the observational test for publication bias, the funnel plot showed a symmetrical distribution (Figure 12). Likewise, Egger's regression test *p* value also indicated the absence of publication bias (*p*=0.59) (Table S6) in Supplementary Materials.

#### 3.3.7. Time Trend Analysis of Carbapenemase-Producing *A. baumannii* in Africa

**Figure 13:**
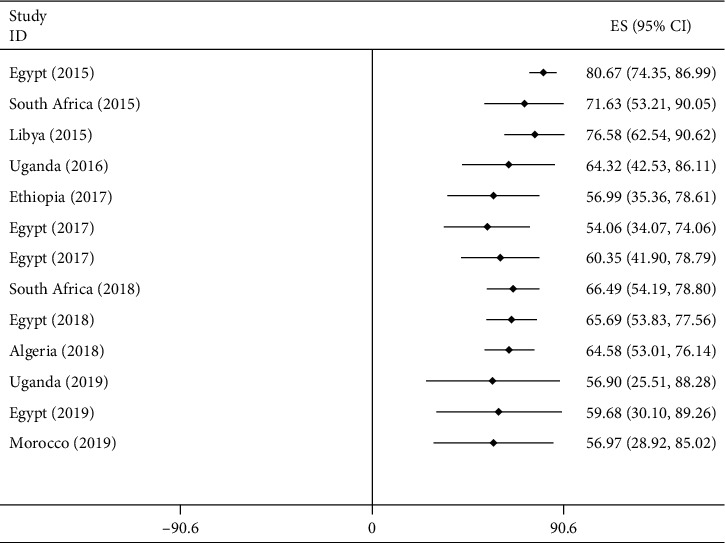
Cumulative meta-analysis of CPAB between 2015 and 2019 in Africa.

According to the cumulative meta-analysis of papers published from 2015 to 2018, CPAB clinical isolates showed high prevalence across the years in the studied African countries (Figure 13).

## 4. Discussion

This systematic review and meta-analysis showed the pooled prevalence of CPPA and CPAB among the clinical specimens in Africa. It also showed the production of different carbapenemase enzymes or detected genes encoding for these enzymes. According to the eligibility criteria of this review, most of the studies of these isolates were conducted in the North Africa region, frequently in Egypt and Algeria. This is consistent with a previous systematic review study among bacterial species producing carbapenemase enzymes in Africa [60].

Although the number of studies varied based on the type of isolates, some of the studies were also done in West, East, and South Africa. But there was no report from Central Africa. This might show that the lack of data on antibiotic resistance in Africa is still a problem for developing and applying evidence-based infection control and prevention measures. Moreover, it might impede patient care and public health, leaving the population vulnerable.

The individual studies included in this systematic review and meta-analysis showed a wide variation on the prevalence of CP isolates in Africa, depending on the bacterial species and geographical region. Moreover, the review also revealed that the CP isolates were more distributed in North African countries. These differences could result from or be attributable to irregular and varied use of antibiotics, low quality of personal hygiene, and inadequate environmental cleaning and infection control policies.

The current review also showed that the pooled prevalence of CPPA was 21.96%. According to this review, VIM carbapenemases were highly prevalent carbapenemase type among *P. aeruginosa* isolates in Africa. This is the same with systematic review and meta-analysis on MBL *P. aeruginosa* conducted in Iran [61]. Moreover, VIM-producing isolates of *P. aeruginosa* had been reported worldwide, including European countries [62] and some countries of Asia [63, 64]. In this review, VIM carbapenemases were widely distributed in most African regions such as in North, East, and West African countries. All types of carbapenemases among *P. aeruginosa* isolates were found only in Egypt.

According to the findings of this meta-analysis, the higher pooled prevalence of CPAB was observed in Africa. This showed that carbapenemase production among *A. baumannii* infection developed fast in African countries. This is also consistent with reviews that showed the fast increasing trend of carbapenem resistance among clinical *A. baumannii* isolates worldwide [65, 66]. This might lead to a “post-antibiotic era” for A*. baumannii* infection in the near future if an immediate action is not taken by the responsible bodies.

The first case of the OXA-type enzyme was reported from a clinical *A. baumannii* isolate detected in Scotland in 1985. It was initially named ARI-1 (*Acinetobacter* resistant to imipenem) [67] and renamed OXA-23 after sequencing [68]. According to this review, the OXA-23 was the predominant carbapenemases in the African *A. baumannii* isolates. This is not surprising, since OXA-23 has been found around the world [69–75]. Importantly, in Africa (particularly in North Africa), studies showed that *bla*OXA-23 is associated with IS*Aba1* elements [13, 31, 76] and predominantly belongs to clone ST2 [22, 23, 31, 76–78], which may enable rapid transmission among OXA-23-producing *A. baumannii* clinical isolates.

Metallo-beta-lactamase (MBL) was detected as a commonly prevailing carbapenemase in Africa among CPPA and CPAB bacterial isolates. This is consistent with other reviews that were conducted globally [79, 80]. MBL genes are usually located in transferable genetic elements such as plasmid and integrons along with other antibiotic resistance genes. Therefore, dissemination of strains harboring MBL genes is of crucial importance, and appropriate measures should be taken into consideration by infection control programs [81].

The current review also showed the emergence of isolates coharboring genes encoding more than one class of carbapenemases in Northern, Eastern, and Western parts of African regions, but mostly in the North Africa region. Concomitant of *bla*OXA-23 + *bla*NDM frequently among *A. baumannii* clinical isolates was observed, particularly in Algeria, [28], Egypt [31, 33, 40], Morocco [45], and Uganda [51]. Moreover, *P. aeruginosa* isolates coharboring genes encoding more than one carbapenemase were seen in different studies conducted in Egypt [11, 33, 34, 37, 41–43] and Sudan, [48, 49]. The emergence of such resistant strains represents a significant threat, not only to these countries but also to the whole of Africa and then to the world, especially as the dissemination of resistance genes is hastened by high rates of immigration and tourism [82–84].

Lastly, the time trend analysis for each of the bacterial isolates included in this review also showed that the prevalence of CPPA relatively increased over time in Africa. However, the prevalence of CPAB clinical isolates showed higher prevalence across the years in Africa.

This review has some limitations: the first was considerable heterogeneity of studies included for the estimation of pooled prevalence of CPPA and CPAB. Therefore, to limit the influence of the study heterogeneity, the random-effects model of DerSimonian and Laird [85] was implemented in the meta-analyses, and subgroup analyses were also performed. Second, studies with different target populations, for example, neonates, adults, or transplant patients, were selected which might have an effect on the study. Third, since data for most parts of the region were not available, these findings might not represent the whole of Africa.

## 5. Conclusion and Recommendations

This review showed the pooled estimate prevalence of CPPA and CPAB across the African continent. It also revealed a high pooled prevalence of CPAB clinical isolates in Africa which needs an urgent action by the responsible bodies such as WHO and FMOH, before the problem becomes totally uncontrollable. Moreover, the emergence of concomitant carbapenemase, OXA-23 + NDM, is in an alarming condition that needs further control and studies.

## Figures and Tables

**Table 1:** Characteristics of studies reporting CPPA and CPAB isolates in Africa from 2007 to 2018 G.C.

Country (Ref.)	Year of sampling	Organism	Number of CP isolates per		Number of isolates with genes encoding carbapenemase enzymes						
			Total isolates	CR	OXA(N)	NDM(N)	VIM(N)	IMP(N)	KPC(N)	Others(N)	Coproducers(N)
Algeria [13]	2008–2014	*A. baumannii*		92/94	OXA23(63) OXA24(16)	10					
Algeria [22]	2015	*A. baumannii*	6/12	6/6	OXA23(6)						
		*P. aeruginosa*	0/9	0/2							
Algeria [23]	2013–2015	*A. baumannii*	7/7	—	OXA23(5)	2					NDM + OXA58(1)
		*P. aeruginosa*	2/18	—							
Algeria [24]	2012-2013	*P. aeruginosa*	0/80	0/15							
Algeria [25]	2014-2015	*P. aeruginosa*	7/31	7/15			7				
Algeria [26]	2009-2012	*P. aeruginosa*	2/89	2/35			2				
Algeria [27]	2015-2016	*P. aeruginosa*	16/188				26				
Algeria [28]	2011–2013	*A. baumannii*	—	47/47	OXA23(26) OXA24(10)	4					NDM + OXA23(7)
Egypt [29]	2015	*P. aeruginosa*	9/70	9/20			9				
Egypt [30]	2014–2016	*A. baumannii*	266/280	266/268				134			IMP + SIM + GIM(120)
											IMP + SIM(12),
Egypt [31]	2012-2013	*A. baumannii*	121/150	121/131	OXA23(62)	6					OXA23 + NDM(53)
Egypt [32]	2013	*A. baumannii*	17/43	17/23	OXA48(13)	3					OXA48 + NDM(1)
		*P. aeruginosa*	3/75	3/13	OXA48(2)	1					
Egypt [33]	2017	*A. baumannii*	30/50	30/30						GES(1)	OXA23 + NDM(15) OXA23 + GES(9) NDM + GES(3) OXA23 + NDM + GES(1)
		*P. aeruginosa*	21/43	21/22		4	5			GES(3)	VIM + GES(4) VIM + IMP(1) IMP + GES(2) NDM + IMP(1) NDM + VIM(1)
Egypt [34]	2014	*P. aeruginosa*	4/7	—			2				VIM + OXA48(2)
Egypt [12]	2013-2014	*A. baumannii*	—	2/7		2					
		*P. aeruginosa*	—	3/15		3					
Egypt [35]	2013-2014	*P. aeruginosa*	30/175	30/30				11		SPM(19)	
Egypt [36]	2011-2012	*P. aeruginosa*	51/122	51/56	OXA10(20)	2	28	1			
Egypt [37]	2014-2015	*P. aeruginosa*	28/33	28/32	OXA10(2)		13				VIM + OXA10(13)
Egypt [38]	2013	*A. baumannii*	—	34/40	OXA23(19) OXA58(1) OXA24(3)					GES(10)	OXA23 + 58+GES(1)
Egypt [11]	2011-2012	*P. aeruginosa*	—	29/48			26	1			VIM + NDM(2)
Egypt [39]	2015	*A. baumannii*	—	48/50	OXA23(48)						
Egypt [40]	2015-2016	*A. baumannii*	53/59	53/53	OXA23(40)	1					OXA23+NDM(4) NDM + OXA58(2) VIM + OXA23(4) VIM + NDM + OXA23(2)
Egypt [41]	2015-2016	*P. aeruginosa*	13/114	13/14			4	1		GIM(4)	VIM + IMP(4)
Egypt [42]	2016–2018	*P. aeruginosa*	26/150	—			7	1		GIM(5) SPM(3)	IMP + GIM(3) IMP + VIM + SPM(1) IMP + SPM(2) IMP + GIM + SPM(1) IMP + VIM(1) VIM + GIM + SPM(1) VIM + GIM(1)
Egypt [43]	2013-2014	*P. aeruginosa*	25/147	25/39						GIM(9) SPM(1) SIM(9)	GIM + VIM(2) SPM + SIM(4) VIM + SPM(1) IMP + VIM + GIM + SPM(1)
Libya [44]	2013-2014	*P. aeruginosa*	19/24	19/21			19				
		*A. baumannii*	22/25	22/22	OXA23(21)						OXA23 + OXA48(1)
Morocco [10]	2012–2014	*P. aeruginosa*	2/68	2/25			2				
Morocco [45]	2015–2016	*A. baumannii*	20/81	20/23	OXA23(4)					GES(3)	OXA23 + GES(8) GES + NDM + OXA23(3) NDM + GES(1) NDM + OXA23(1)
Tunisia [46]	2015-2016	*A. baumannii*	—	3/3	OXA23(3)						
Tunisia [47]		*P. aeruginosa*	14/64	14/36			14				
Sudan [48]	2015-2016	*P. aeruginosa*	23/54	—		1	8	4			VIM + IMP(1) VIM + NDM(6) IMP + NDM(2) VIM + NDM + IMP(1)
Sudan [49]	2015	*P. aeruginosa*	—	5/14				1			IMP + KPC + VIM(2) KPC + IMP(2)
		*A. baumannii*	—	25/37			6				IMP + KPC + VIM(17) KPC + VIM(2)
Ethiopia [50]	2014-2015	*A. baumannii*	3/13	3/3		3					
Uganda [51]	2007–2009	*P. aeruginosa*	9/42	9/10			5	4			
		*A. baumannii*	7/29	7/9	OXA23(4) OXA23 + NDM(1)						OXA58(1) OXA23 + OXA58(1)
Uganda [52]	2015–2017	*P. aeruginosa*	25/1077	25/29			25				
		*A. baumannii*	21/26	21/448	OXA23(10) OXA24(8)		13				OXA23 + OXA24(5)
Tanzania [53]	2010-2011	*P. aeruginosa*	8/90	8/8			8				

Ghana [54]	2012–2014	*A. baumannii*	—	9/31		9					
		*P. aeruginosa*	—	9/51		2	7				
Nigeria [55]	2013–2015	*P. aeruginosa*	5/200	5/22			5				
South Africa [56]	2013-2014	*A. baumannii*	136/141	136/137	OXA23(132) OXA58(4)						
South Africa [57]	2015	*A. baumannii*	—	24/24	OXA23(24)						
South Africa [58]	2008	*A. baumannii*	60/97	—	OXA23(57) OXA48(3)						
South Africa [59]	2013	*P. aeruginosa*	—	11/15						GES(7)	GES + VIM(4)

## Supplementary Materials

Supplementary Materials Figure S1: forest plot for the pooled prevalence of CPPA among CRPA isolates in Africa. Figure S2: the subgroup analysis showed the pooled prevalence of CPPA isolates in Africa by region. Table S3: Egger's regression test for publication bias of CPPA. Figure S4: forest plot for the pooled prevalence of CPAB among CRAB isolates in Africa. Figure S5: the subgroup analysis showed the pooled prevalence of CPAB isolates in Africa by Region. Table S6: Egger's regression test for publication bias of CPAB.
