# Supplementary material for: Carbapenemase-Producing Non-Glucose-Fermenting Gram-Negative Bacilli in Africa, Pseudomonas aeruginosa and Acinetobacter baumannii: A Systematic Review and Meta-Analysis
Source: Int J Microbiol. 2020 Nov 3;2020:9461901. doi: 10.1155/2020/9461901 (PMC7658691; doi:10.1155/2020/9461901)

**Supplementary files**

**
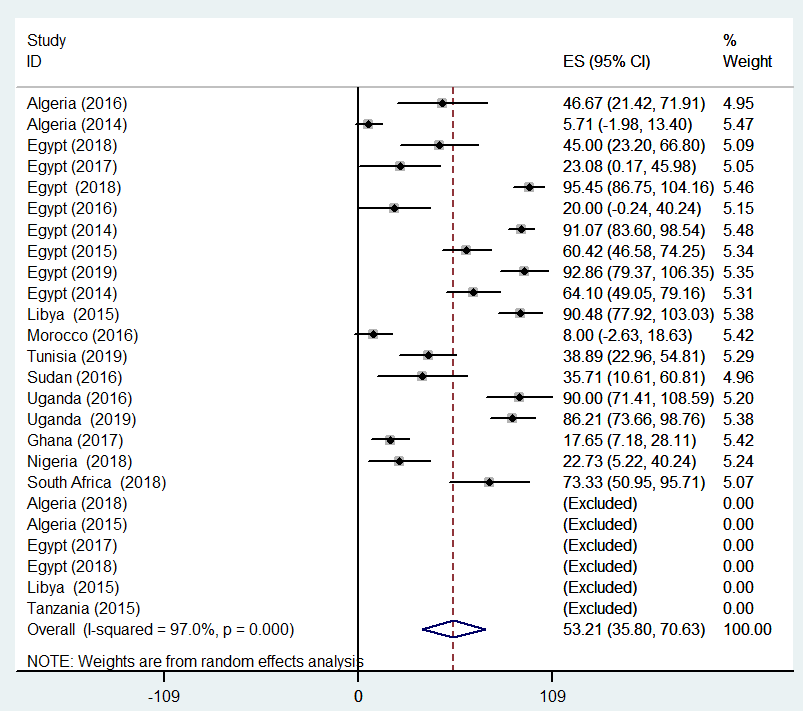
**

**Figure S1:** Forest plot for pooled prevalence of CPPA among CRPA isolates in Africa


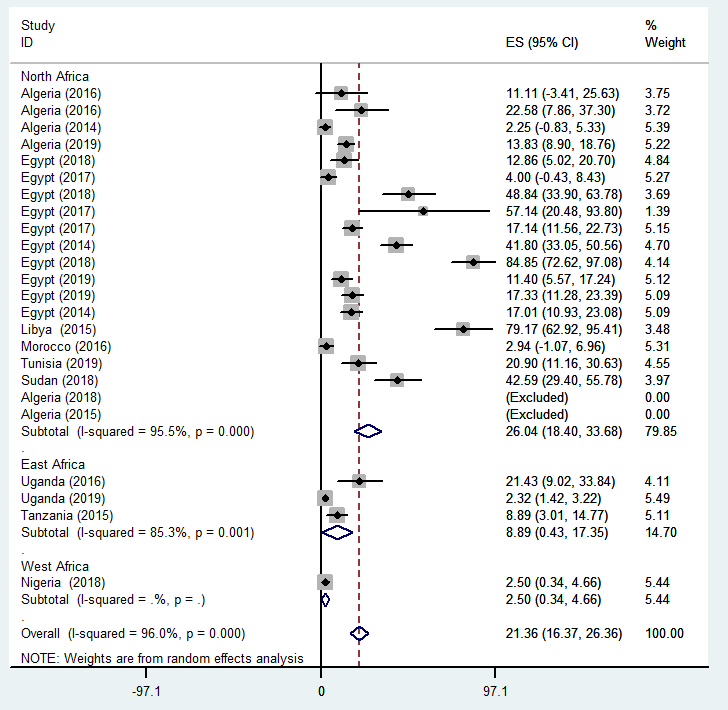


Figure S2: The subgroup analysis showed the pooled prevalence of CPPA isolates in Africa by Region

**Table S3:** Egger’s regression test for publication bias of CPPA


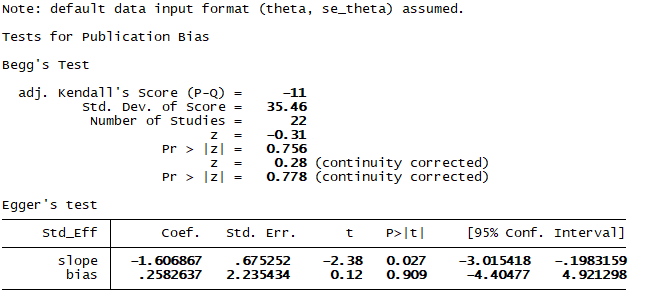


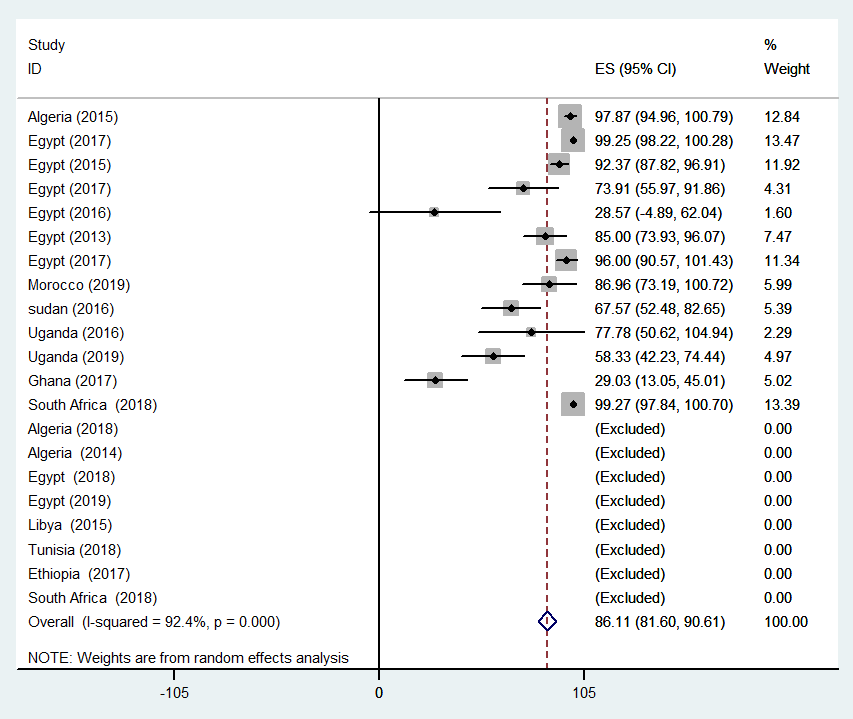


**Figure S4:** Forest plot for pooled prevalence of CPAB among CRAB isolates in Africa


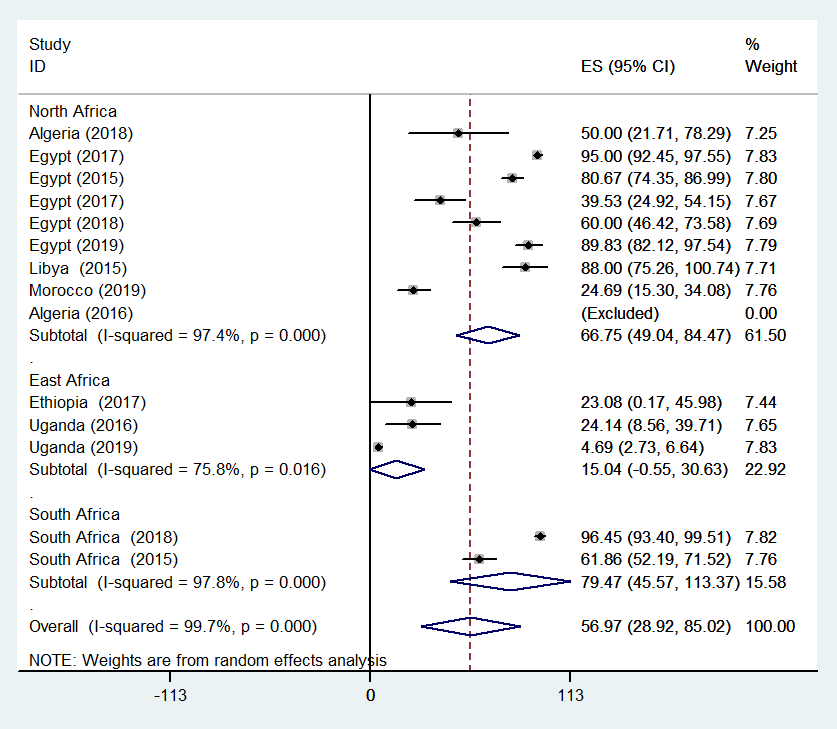


Figure S5: The subgroup analysis showed the pooled prevalence of CPAB isolates in Africa by Region

**Table S6:** Egger’s regression test for publication bias of CPAB


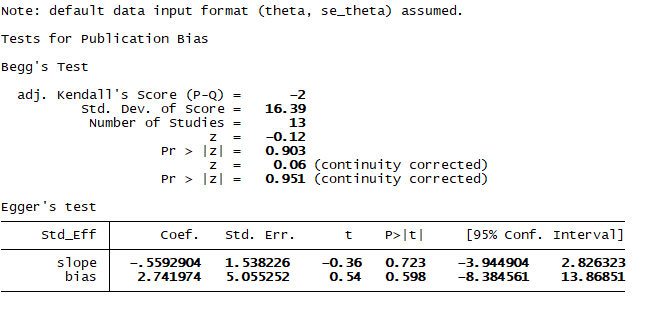

Supplement: Supplementary Materials — Figure S1: forest plot for the pooled prevalence of CPPA among CRPA isolates in Africa. Figure S2: the subgroup analysis showed the pooled prevalence of CPPA isolates in Africa by region. Table S3: Egger's regression test for publication bias of CPPA. Figure S4: forest plot for the pooled prevalence of CPAB among CRAB isolates in Africa. Figure S5: the subgroup analysis showed the pooled prevalence of CPAB isolates in Africa by Region. Table S6: Egger's regression test for publication bias of CPAB. [file 9461901.f1.docx]
